# Effect of Hypertonic Saline 5% on Early Graft Function and Urinary Interleukin 18 and Neutrophil Gelatinase-Associated Lipocalin in Deceased-Donor Kidney Transplantation

**Published:** 2017

**Authors:** Mojtaba Mojtahedzadeh, Farhad Etezadi, Gholamreza Pourmand, Amir Hossein Najafi Abrandabadi, Javad Motaharinia

**Affiliations:** a *Department of Pharmacotherapy, Faculty of Pharmacy, Tehran University of Medical Sciences, Tehran, Iran.*; b *Department of Anesthesiology & Critical Care, Sina Hospital, Tehran University of Medical Sciences, Tehran, Iran. *; c *Urology Research Center, Tehran University of Medical Sciences, Tehran, Iran.*

**Keywords:** Hypertonic saline, Deceased donor, Kidney transplantation, Delayed graft function, Neutrophil gelatinase-associated lipocalin and interleukin 18

## Abstract

Ischemia reperfusion injury (IRI) is one of the main causes of delayed graft function (DGF) in deceased-donor kidney transplantation (DDKT). Evidences suggest that hypertonic saline (HS) has beneficial effects on IRI. The objective of the present study is to determine the effect of intraoperative HS, on graft function and urinary biomarkers of interleukin 18 (IL-18) and neutrophil gelatinase-associated lipocalin (NGAL), in patients with DDKT. The design of the study is a randomized, open-label, pilot trial in patients with DDKT. The intervention of the study is administration of 4 mL/kg HS, 5% before graft reperfusion. The primary endpoint was DGF. Fifty-eight (58) adult patients were randomized (HS, n = 32; control, n = 26). There were no significant differences between the two groups in terms of recipient, donor, and transplant characteristics. The rate of DGF was 20% in the HS group compared with 31.8% in the control group (Relative risk 0.63; 95% CI 0.23–1.67; *P* = 0.36). Serial serum creatinine in the first two days after surgery in addition to urine volumes during the first day after transplantation was significantly different in the HS group (*P* ≤ 0.05). The urinary NGAL and IL-18 were significantly lower in HS vs. control, at 24 h after transplantation (*P* ≤ 0.05). The frequency of adverse reactions was similar between groups. This study did not show any significant benefits from HS administration immediately before allograft reperfusion in terms of reducing DGF, serum creatinine at hospital discharge or length of hospital stay in deceased-donor kidney transplant patients.

## Introduction

Kidney transplantation has become a preferred treatment for most patients with end-stage renal disease and is superior to dialysis in terms of quality of life and long-term mortality risk (1). Ischemia and reperfusion during kidney transplantation initially causes endothelial and tubular epithelial cells injuries. The injuries may be so severe that they lead to acute kidney injury (AKI) and delayed graft function (DGF) (2). The injury mechanism is complex and not well understood yet. However, it is well known that graft ischemia reperfusion injury (IRI) is an antigen-independent inflammatory condition, first mediated by innate immunity (3). 

Attempts to reducing graft IRI include avoiding hypovolemia and nephrotoxic drugs, minimizing warm and cold ischemia times, using vasodilator, immunosuppressive and anti-inflammatory drugs, as well as antioxidant therapy (2). Although many modalities including therapeutic gases, anti-oxidant therapy, immunosuppressive and anti-inflammatory drugs, and small interfering RNA have been studied for attenuation of renal IRI, the results are contradictory (4). 

Hypertonic saline now is widely used as a resuscitation fluid during critical illness because of its beneficial hemodynamic properties, such as rapid expansion of intravascular volume, reduction of endothelial and tissue edema that improves microcirculation, and increased myocardial contractility (5, 6). Besides the improvement in hemodynamic parameters, new findings have suggested that HS modulates various functions of immune cells such as degranulation, ROS production, cytokine release and adhesion molecules expression (7). Several studies on animal models have shown that administration of HS immediately before tissue reperfusion can reduce IRI (8-11). However, HS has some drawback including hyperchloremic metabolic acidosis, hypernatremia and fluid overload (12, 13). 

Interleukin 18 (IL-18) and neutrophil gelatinase-associated lipocalin (NGAL), play a pathogenic and protective role for kidney IRI, respectively. Recently, IL-18 and NGAL were proposed as promising biomarkers, hence their measurement in urine samples can be used to detect DGF in kidney transplant patients (14).

Considering the aforementioned facts, HS could be beneficial for attenuating IRI, a key factor in the development of DGF. To the extent of our knowledge, HS has not been studied in kidney transplantation. Therefore, we carried out a pilot randomized clinical trial to evaluate the effect of bolus administration of HS, at the time of graft reperfusion on the early kidney allograft function and urinary biomarkers of kidney injury, including IL-18 and NGAL.

## Experimental

*Patients*

All consecutive adult deceased donor kidney transplant recipients at Sina hospital (Urology Research Center, Tehran University of Medical Sciences), between October 2013 and September 2014 were approached to participate in this randomized, open-label, pilot trial. The study was approved by the Ethics committee of Tehran University of Medical Sciences and registered under the Iranian registry of clinical trials (IRCT2013122815841N19). The inclusion criteria included a first transplantation, deceased-donor kidney transplantation, recipients’ ages between 18 to 65 years, left ventricular ejection fraction >30%, panel reactive antibody <20%, and donor serum creatinine <2 mg/dl. The exclusion criteria included severe acidosis (pH ≤7.2 and/or serum bicarbonate ≤10 mEq/l and/or Base excess (BE) ≤ -15 mEq/l), electrolyte abnormality (k<3.5 mEq/dl or Na<125 mEq/dl or Na>145 mEq/dl), blood product transfusion, colloidal fluid administration, graft anastomosis bleeding or thrombosis.

*Intervention*

A computer-generated list with three random-block sizes (4, 8 or 12) was used to randomize patients on a 1 to 1 ratio to HS 5% or normal saline (NS) solutions. The patients in the HS and NS groups received 4 mL/kg of HS 5% or NS within 15 minutes starting 10 min before graft reperfusion. 

Normal saline approximately at a dose of 20 mL/kg/h administered to all the patients as intra-operative fluid therapy and titrated to keep the central venous pressure between 10 and 15 cm H_2_O and systolic blood pressure more than 140 mmHg at graft reperfusion time and thereafter. In both groups, patients’ acid-base states were measured after induction of anesthesia, during the vascular anastomosis procedure, before administration of intervention solutions, and after graft reperfusion. As our transplantation protocol, all patients received 5 mg/kg of furosemide just before graft reperfusion. All the transplantation surgeries were performed by the same transplant surgeon.

The immunosuppressive regimen involved induction with daily ATG at a dose of 1 mg/kg for 5 days, followed by triple-drug immunosuppressive therapy. All patients received methylprednisolone 500 mg during transplantation surgery, 250 and 100 mg on the second and third day post-transplant which was then converted to prednisone and tapered to 5 mg daily for several months. Mycophenolate mofetil was administered at a dose of 1 g twice daily, starting the day of transplantation. Cyclosporine administered after transplantation, when serum creatinine had fallen to an acceptable level, at a dose of 6 mg/kg/day, as divided twice daily.

**Figure 1:**
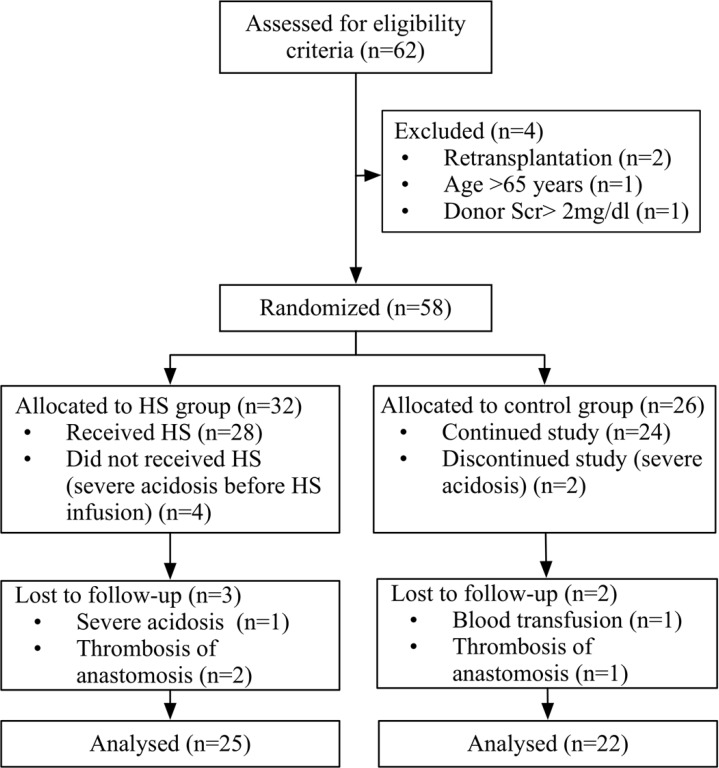
CONSORT flow chart for study group disposition

**Figure 2:**
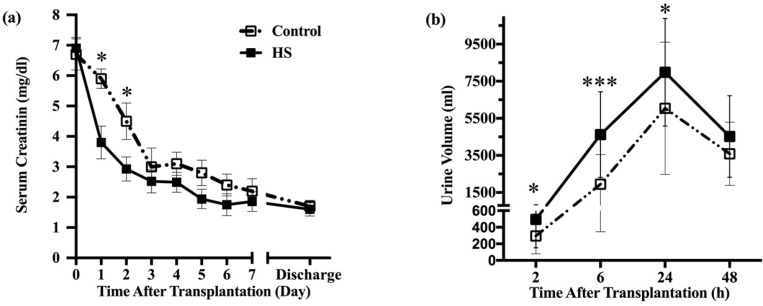
Comparison of graft function in HS and control groups. (a) Those patients treated with HS; the serum creatinine levels were significantly lower at postoperative day one to day three. Subsequent serum creatinine values continued to be lower for the HS group patients, but the differences no longer achieved statistical significance at day four today 7. (b) Patients who received HS had significantly higher urine volume compared with control group at two-, six- and twenty four-hour period after transplantation. However, urine volume did not differ between groups at second-day post-transplant. HS: hypertonic saline. * P < 0.05, ** P < 0.01, *** P < 0.001

**Table 1:** Recipient, donor and Transplant characteristics of patients

**Recipient characteristics**					**Control (n = 22)**	**Hypertonic saline (n = 25)**	**P-value**
	Age (years)				46 (14) *	41 (10)	0.12
	Sex (% male)				60*	72	0.52
	BMI (kg/m^2^)				24.3 (6.5)	22.1 (4.2)	0.18
	Cause of end-stage renal disease (%)						
				Diabetes	15	20	0.27
				Hypertension	33	20	
				Obstructive uropathy	14	8	
				Infection	9.5	12.5	
				Polycystic kidney	9.5	4	
				others	19	34.5	
	Time on pre-transplant dialysis (month)**				15 (63)	24 (57)	0.25
	Last dialysis session before transplant (day) **				1 (2)	1 (3)	0.7
	Serum creatinine before transplantation (mg/dL)				6.8 (2)	6.54 (1.73)	0.25
Donor characteristics							
	Age (years)				34(12)	30 (12)	0.1
	Sex (% male)				76	64	0.52
	Serum creatinine (mg/dL)				1.2 (0.35)	1.26 (0.3)	0.34
	Cause of brain death (%)						
			CVA		30	8	0.09
			Head trauma		50	66	
			Post CPR		10	18	
			Drug Intoxication		10	4	
			Brain tumor		0	4	
Transplant characteristics							
	Cold ischemic time (h)				4.2 (1.1)	4.26 (1.1)	0.8
	Warm ischemic time (min)				43 (6)	49 (10)	0.2
	MAP at the time of graft reperfusion (mmHg)				103 (11)	110 (10)	0.1
	Acid-base state before HS administration						
		Arterial pH			7.34 (0.06)	7.38 (0.07)	0.053
		Base excess (mEq/L)			-7.5 (-3.7)	-6.5 (-3.3)	0.32
		Serum bicarbonate (mEq/L)			15 (4)	18 (5)	0.56
		PaCO2 (mmHg)			37 (5)	35(7)	0.43
	Acid-base state 10 min after graft reperfusion						
		Arterial pH			7.26 (0.05)	7.31 (0.01)	025
		Base excess (mEq/L)			-10.7 (-3.2)	-7.7 (-3.5)	0.016
		Serum bicarbonate (mEq/L)			15 (5)	15.9 (3)	0.7
		PaCO2 (mmHg)			37 (5)	37 (3)	0.9
	Saline 0.9% volume (mL/kg)				57 (23)	51 (19)	0.73
	Serum Na before HS administration (mEq/L)				-	141 (4)	-
	Serum Na at the end of HS administration				-	147 (6)	-

BMI: Body mass index, CVA: Cerebrovascular accident, CPR: Cardiopulmonary resuscitation, MAP: Mean arterial pressure, HS: Hypertonic saline 5%, ATG: Anti-thymocyte globulin.

* Data presented as mean (SD) or percentage.

** Data presented as median (range)

**Table 2:** Comparison of study findings between control and Hypertonic saline groups

**Primary and secondary endpoints**				**Control (n = 22)**	**Hypertonic saline (n = 25)**	**P-value**
	Graft function (%)					
		Immediate		62	76	0.506
		Slow		9.5	12	
		Delayed		28.5	12	
	Urinary IL-18/Cr (pg/mg) *					
			2h post-transplant	2681 (1348)	3 1742 (957)	0.039
			24h post-transplant	4579 (1942)	2061 (1173)	0.004
			48h post-transplant	738 (1426)	2216 (1240)	0.097
	Urinary NGAL/Cr (ng/mg) *					
		2h post-transplant		10153 (5532)	19329 (5725)	0.19
		24h post-transplant		9875 (4476)	6359 (3750)	0.017
		48h post-transplant		9063 (4182)	8471 (3582)	0.36
	Incidence of acute rejection (%)			35	24	0.5
	Length of hospital stay (day) **			15 (13)	13 (14)	0.24

IL-18: Interleukin 18, NGAL: Neutrophil gelatinase-associated lipocalin. Cr: Creatinine.

* Data presented as mean (SD) or percentage.

** Data presented as median (range)

**Table 3:** Clinical characteristics of patients with different graft function

**Recipient characteristics**			**IGF**	**SGF**	**DGF**	**P-value**
	Age (years)		43 (12) *	43 (13)	40 (14)	0.89
	Sex (% male)		71*	40	76	0.4
	BMI (kg/m^2^)		22.1 (5.3)	21.4 (3.5)	23.1 (3.9)	0.26
	Time on pre-transplant dialysis (month)**		20 (56)	42 (24)	12 (64)	0.037
	Last dialysis session before transplant (day) **		1 (3)	1 (1)	1(2)	0.27
	Serum creatinine before transplantation (mg/dL)		6.45 (1.76)	7.71 (1.97)	7.25 (1.82)	0.25
Donor characteristics						
	Age (years)		30(12)	29 (9)	34 (13)	0.54
	Sex (% male)		62	100	78	0.12
	Serum creatinine (mg/dL)		1.18 (0.53)	1.26 (0.47)	1.27 (0.26)	0.7
	Cause of brain death (%)					
		CVA	25	30	35	0.14
		Head trauma	43	50	30	
		Post CPR	14	10	0	
		Drug Intoxication	0	10	0	
		Brain tumor	18	0	35	
Transplant characteristics						
	Cold ischemic time (h)		4.34 (1.24)	4.24 (0.25)	4.33 (1.13)	0.9
	Warm ischemic time (min)		45 (7)	47 (9)	43(7)	0.5
	ATG- based immunosuppression induction (%)		47	60	55	0.8
Biomarkers						
	Urinary IL-18/Cr (pg/mg)					
		2h post-transplant	1737 (1258)	2709 (1612)	3147 (2349)	0.002
		24h post-transplant	1942 (1113)	2342 (1313)	3768 (2077)	0.001
		48h post-transplant	2132 (1171)	2596 (1547)	2725 (1883)	0.083
	Urinary NGAL/Cr (ng/mg)					
		2h post-transplant	7649 (4091)	8593 (4927)	8883 (4578)	0.078
		24h post-transplant	7518 (3724)	10675 (5432)	12247 (6137)	0.0047
		48h post-transplant	6259 (3941)	9637 (5274)	11923 (6483)	0.001

IGF: Immediate graft function, SGF: Slow graft function, DGF: Delayed graft function, BMI: Body mass index, CVA: Cerebrovascular accident, CPR: Cardiopulmonary resuscitation, ATG: Anti-thymocyte globulin, IL-18: Interleukin 18 and NGAL: Neutrophil gelatinase-associate lipocalin, Cr: Creatinine.

* Data presented as mean (SD) or percentage.

** Data presented as median (range)

*Study endpoints*

The primary endpoint was DGF. DGF defined as the need for dialysis within the first week after transplantation. In addition, the early functioning grafts were divided into two groups, slow graft function (SGF) and immediate graft function (IGF). SGF defined as serum creatinine level above 2.5 mg/dl on the fifth day of kidney transplantation and the remaining was considered as IGF (15). The secondary endpoints consisted of degree of acute kidney injury (AKI) assessed using urinary NGAL and IL-18 levels measured at multiple time points, serum creatinine, urine volume, incidence of acute rejection (AR), duration of hospital stay and adverse events.

To measure urinary biomarkers, 5 mL urine samples were collected directly from the patients› urinary catheter at 2, 24, and 48 h after graft reperfusion. Urine samples were centrifuged at 5000×g for 5 min at room temperature and stored at 80 °C. Interleukin 18 and NGAL levels were measured using Human IL-18 ELISA Kit and Human NGAL ELISA Kit purchased from Shanghai Crystal Day Biotech Co, China. The coefficient of variation (inter-assay variability) for both biomarkers was less than 12%. Urinary biomarkers were normalized to urinary creatinine concentration in order to account for the differences in the glomerular filtration rate and resultant urinary flow.

Serum creatinine was measured daily until discharge and urine volume recorded at two, six, and 24 h after transplantation, then daily within the first week after transplantation.

Baseline recipient characteristics such as age, sex, body mass index, length of hemodialysis, time of last hemodialysis session before transplant surgery and donor characteristics of age, sex, serum creatinine, warm, and cold ischemia time recorded for all patients.

*Statistical Analysis*

Patient demographic information and baseline characteristics were summarized using descriptive statistics. The recipient, donor, and transplant parameters were compared using unpaired two-sample *t*-tests or cross-tables and Fisher’s exact test. If the statistical assumptions for parametric analysis of interval variables were not met, Mann–Whitney’s two-independent samples test was used. Different variants of multiple measurements were separately analyzed using repeated measurement analysis. The rates of DGF and AR were compared using cross-tables and Fisher’s exact test. A value of P < 0.05 was considered statistically significant. Analyses were conducted using the SPSS software package version 19. We used the method described by Cocks. et al to estimate the sample size for this pilot randomized trial (16). Assuming HS may decrease the incidence of DGF from 25% to 15%, type-1 error of 5%, study power of 80%, and using an 80% one-sided confidence interval, the sample size was 29 patients in each group. This sample size was chosen to provide preliminary data to inform design of future randomized controlled trial. 

## Results

From October 1, 2013, through September 31, 2014, sixty-two (62) potential deceased-donor kidney transplant recipients were identified and screened for eligibility criteria (Figure 1). Four patients were excluded because of previous transplant (n = 2), donor serum creatinine of more than 2 mg/dL (n = 1) and age of more than 65 years (n = 1). The remaining fifty-eight (58) patients were randomized to HS (n = 32) or control groups (n = 26).

In the intra-operative period, five patients in the HS group were excluded with severe acidosis before (n = 4) or after HS administration (n = 1). Three patients in the control group were excluded with severe acidosis (n = 2) or blood product transfusion (n = 1). In the follow up period, one patient in the control group and two patients in the HS group were excluded because of graft vein thrombosis. Twenty-five (25) patients in the HS group and twenty-two (22) patients in the control group, were included from the statistical analysis.

The baseline characteristics of the recipient and donor are summarized in Table 1. There were no differences in baseline recipient or donor characteristics. Also, there were no statistically significant differences between the groups in the cold ischemic time, mean arterial pressure, arterial pH and base excess.

The average cold ischemia time was 4.91 h (range, 2.9–6.8 h) in the HS group and 4.72 h (range, 3–6.5 h) in the control group. Patients in the HS group received 4 mL/kg HS 5% as bolus administration. Although the baseline acid-base state did not differ between the two groups, the arterial blood pH and BE 10 min after graft reperfusion, were lower in the control group compared to HS group (7.29 (0.03) vs 7.34 (0.04), P = 0.25). In addition, mean of BE was statistically lower in the control group (-8.5 (-3.1) vs -10.2 (-3.4), *P* = 0.02). 

The rate of DGF, SGF, and IGF occurrences after transplantation are shown in Table 2. The incidence of DGF did not significantly differ between both groups, though, the rate of DGF was slightly lower in the HS group (Relative risk 0.63; 95% CI 0.23–1.67; *P* = 0.36). Generally, the donor and recipient characteristics were not different among patients with various graft functions (Table 3). Multivariate analysis of our data revealed that recipient sex, recipient age, donor age, cause of brain death, and arterial blood gas parameters were not independent predictors of DGF. The incidence of AR was lower in the HS group, but this was not statically significant (24% vs. 35%, *P*=0.5). However, the incidence of AR was lower in the subgroup of patients with IGF (*P*=0.043).

Early post-transplant graft function in terms of serum creatinine and urine volume significantly improved in patients who received HS (Figure 2). However, serum creatinine was not different between groups at the time of hospital discharge (1.6 vs. 1.7 mg/dl). The length of hospital stay did not differ between the HS and control groups. However, the trend was toward shorter duration in the HS treated patients (13 vs. 15 days, *P*=0.24).

Urine IL-18 to creatinine ratio (IL-18/Cr) and NGAL to creatinine ratio (NGAL/Cr) in the HS group were lower at all sampling times. These differences for urine IL-18/Cr at 2 and 24 h and for urine NGAL/Cr at 24 h after graft reperfusion, achieved statistical significance (Table 2). The urinary IL-18/Cr and NGAL/Cr in the patients with DGF were higher than those with SGF or IGF (Table 3). The post HOC analysis showed that the difference for IL-18/Cr levels between patients with DGF and IGF was significant at 2 h (*P*=0.002) and 24 h after graft reperfusion (*P*=0.001). In addition, the difference for NGAL/Cr levels between patients with DGF and IGF was significant at 24 h (*P*=0.0047), and 48 h after graft reperfusion (*P*=0.001). 

The infusion of HS was generally well tolerated. Only, two patients experienced severe hyperchloremic metabolic acidosis after HS administration. Nonetheless, no clinically significant electrolyte or hemodynamic abnormalities such as pulmonary edema and cardiac arrhythmias were observed in the patients. 

## Discussion

This is the first randomized, case-controlled, trial that evaluated the efficacy of HS in improving early graft function, in patients receiving kidney transplantation from a deceased donor. Our findings did not show a significant reduction in DGF occurrence after HS administration starting 10 min prior to allograft reperfusion. Serum creatinine at hospital discharge and length of hospital stay were not significantly different between HS and control groups.

In this study, we observed that HS administration shortly prior graft reperfusion improved early graft function in terms of urine output and serum creatinine. These results may be explained by osmotic and hemodynamic effects of HS. The endothelial cell (EC) injury and swelling initiated during the ischemia phase is the key to the microcirculatory disturbance after reperfusion (17). By establishing an osmotic gradient, HS reduces EC swelling and facilitates microvascular circulation (7). Also, the increased vasomotion activity of microvascular is offered as a possible mechanism for microcirculation improvements by HS (7). Furthermore, mobilization of fluids from intracellular to extracellular compartments by HS and increasing intra-vascular volume might improve renal perfusion (5). 

In our study, HS infusion was at the time of high-dose furosemide administration. Studies in acute decompensated heart failure patient shown that combination of HS and high dose furosemide was associated with increased urine output and enhanced weight loss as well as significantly smaller increase in serum creatinine (18). HS enhances the effects of furosemide by transiently increasing the serum sodium, thereby delivering adequate amount of sodium ions at the level of the tubular lumen of Henle’s loop (19, 20). Furthermore, HS may attenuate the possible harmful effects of neuro-hormonal excitation as well as the i.v. diuretic-related reduction in glomerular filtration rate following high dose furosemide (20). It is possible, by strengthening the natriuretic effect of furosemide as well as maintaining the glomerular blood flow and glomerular filtration rate, HS cause a rapid decline in serum creatinine and increase in urine output. These effects secondary to the initial development of hypertonicity/hypernatremia may, partly, explain the improvement in graft function in terms of urine output and serum creatinine demonstrated in the present study. 

IL-18 and NGAL are early markers of tubular damage that their serum and urine levels reflect degree of tubular damages due to IRI. Urinary levels of these biomarkers were measured to determine whether HS improves allograft function by attenuating tubular damage caused by ischemia-reperfusion, in addition to modulation of renal hemodynamics. Furthermore, these biomarkers were measured to evaluate subtle reduction of kidney damage caused by HS that clinical tests are not able to detect. In this study, the urinary levels of both biomarkers were significantly lower 24 h after reperfusion in patients who received HS. This suggests that HS has been able to reduce the tubular damage. At the end of HS 5% administration in this study, the serum sodium level increased by 5.8 mEq/l (from 141.5 to 147.3 mEq/l) and remains elevated for up to 30 min. the occurrence of hypernatremia/hypertonicity during the first minutes of reperfusion may decrease oxidative stress and immune- mediated IRI. It is demonstrated that an increase of 10 to 20 mOsm/kg in plasma osmolality caused by HS modulates local and systemic inflammatory response (12). However, recent findings suggest that modulatory effect of HS on immune system was not caused only by an increase in osmolality, but also to an electrolyte -specific effect (21, 22). Thiel et al. by using different types of hypertonic media such as mannitol, sucrose and choline chloride showed that HS inhibits b2 integrin expression on neutrophil surface via sodium-dependent events rather than by chloride or its osmolality (21). The study by Hatanaka et al. demonstrated alterations in inflammatory cytokine release by HS occurs by an electrolyte-specific effect in addition to the hyperosmolality (22).

Activation of the innate immune system and oxidative stress during reperfusion are associated with graft IRI (3). A series of factors, including release of pro-inflammatory cytokines by tubular epithelial and endothelial cells, induction of adhesion molecules expression on the ECs, and infiltration of neutrophils play a major role in the damage (3). The beneficial effects of HS can be partly explained by its ability to suppress the various functions of neutrophil including adhesion molecule expression, release of proteolytic enzymes, and production of ROS (7, 12). Endothelial activation increases the adherence of neutrophils promoting capillary congestion and the no-reflow phenomenon (17, 23). By inhibiting the Intercellular adhesion molecule-1 (ICAM-1) and b2 integrin upregulation, HS decreases neutrophil rolling and adherence to ECs, thereby improving microcirculation and vascular permeability (7, 24, 25). Several models of hemorrhagic shock and IRI demonstrated that HS attenuates oxidative stress by decreasing inducible nitric oxide synthase (iNOS) expression and increasing heme oxygenase -1 (HO-1) expression (26, 27).

Although arterial blood pH and BE were reduced after administration of HS, only one patient recorded severe acidosis. Nonetheless, no electrolyte or hemodynamic abnormalities such as pulmonary edema and cardiac arrhythmias were observed in the patients. Therefore, controlled administration of HS regarding patients› acid-base states not only provides them with beneficial effects, but also minimizes such complications as hyperchloremic metabolic acidosis, electrolyte imbalance, and fluid volume overload. 

Limitations: Firstly, the small sample size is the main limitation of this study. Secondly, the incidence of DGF in this study was lower in comparison with other reports that have affected the power of study. Thirdly, the clinical judgment of practitioners on the need for dialysis might overestimate the rate of DGF and enter a possible bias. 

In conclusion, this study did not show any significant benefits from HS administration immediately before allograft reperfusion in terms of reducing DGF, serum creatinine at hospital discharge or length of hospital stay in deceased-donor kidney transplant patients (DDKT). The bolus administration of a single dose of HS shortly before graft reperfusion is safe and associated with a more rapid decrease in serum creatinine, increase in urinary volume and lower levels of biomarkers in patients with DDKT. However, future trials using larger study population is needed to confirm the efficacy and safety of intra-operative administration of HS in kidney transplant patients. 
